# Onset of frictional sliding of rubber–glass contact under dry and lubricated conditions

**DOI:** 10.1038/srep27951

**Published:** 2016-06-13

**Authors:** Ari J. Tuononen

**Affiliations:** 1 Aalto University Department of Mechanical Engineering, PO Box 14300, FI-00076 Aalto Finland

## Abstract

Rubber friction is critical in many applications ranging from automotive tyres to cylinder seals. The process where a static rubber sample transitions to frictional sliding is particularly poorly understood. The experimental and simulation results in this paper show a completely different detachment process from the static situation to sliding motion under dry and lubricated conditions. The results underline the contribution of the rubber bulk properties to the static friction force. In fact, simple Amontons’ law is sufficient as a local friction law to produce the correct detachment pattern when the rubber material and loading conditions are modelled properly. Simulations show that micro-sliding due to vertical loading can release initial shear stresses and lead to a high static/dynamic friction coefficient ratio, as observed in the measurements.

The rubber static friction coefficient μ_s_ contributes, e.g., to the grip of tyres, shoes and windshield wipers, even though research is often focused on the sliding (dynamic) friction coefficient μ_d_. The μ_s_ is not purely a material property of contacting surfaces because it depends on the load, temperature, loading conditions and lubrication^1^,^2^,^3^. Large variations in the measured μ_s_ are often observed in experiments (compared, e.g., to μ_d_), which makes it difficult to reproduce measurement results and characterize the frictional performance of materials. In addition, the transition of a static obstacle into steady sliding motion is not properly understood. The literature contains extensive research on the sliding and rolling friction of rubber^4^,^5^,^6^,^7^,^8^ but few studies on how the frictional sliding of rubber is locally initiated^9^,^10^.

Rubber is an interesting material for friction testing even from a more generic perspective. Rubber is nearly incompressible (Poisson’s ratio υ ≈ 0.5), resulting in clear initial shear stresses that depend on how loads or constraints (vertical and tangential) are applied to the sample. Furthermore, rubber is a very elastic material, which introduces two important aspects in experimental testing involving optical methodologies: large displacements (spatial resolution) and slow detachment at the interface (time resolution). These factors are especially important in high-speed imaging, where the limiting factor is the data flow, not only the spatial or time resolution. Rubber materials also contain filler materials that can be traced by, e.g., digital image correlation (DIC).

The onset of the frictional sliding of a polymethyl methacrylate (PMMA) beam has been studied comprehensively^1^,^11^,^12^,^13^,^14^,^15^; however, only a one-dimensional detachment front pattern and velocity are often evaluated because of methodological limitations. Two-dimensional studies have been completed for an elastomer polydimethylsiloxane (PDMS), where the contact of a glass lens and the smooth rubber surface was determined optically^10^,^16^,^17^,^18^.

In this work, we demonstrate through both experimentation and simulation how initial frictional shear stresses caused by vertical loading define a detachment pattern. The experiments were conducted using a high-speed camera and DIC synchronized with 3-dimensional force measurements to study the detachment process. The material is a tread rubber compound used in the production of tyres; thus, the results have direct practical importance. The simulations were 3-dimensional finite element method studies, and they resulted in detachment patterns similar to those observed in experiments. Our results provide insight into how the μ_s_/μ_d_ ratio can be modified, e.g., to achieve desirable static friction properties for different applications. As an example, an increase in static friction in automotive tyres would be very beneficial because an anti-blocking system (ABS) maintains a relative slip as low as 5–15%; consequently, a fully braked tyre is actually generating a substantial braking force by sticking to the road. Thus, the static friction strongly influences the emergency braking distance and the manoeuvrability of modern cars.

## Results

### Experimental results

**Figure 1:**
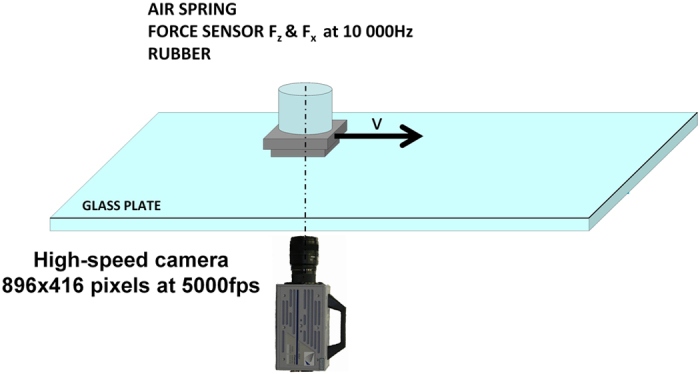
The experimental setup comprising a linear friction tester, a force sensor with rubber sample, a glass substrate and a high-speed camera.

A rubber sample (60 × 60 mm^2^) was pressed against dry and wet glass surfaces in a linear friction tester (Fig. 1). After loading, a longitudinal force was applied to the top surface of the sample. The forces acting on the sample were measured, and the rubber surface movement was high-speed photographed through a transparent glass plate. The high-speed camera image sequences were analysed by DIC to investigate the detachment process.

### Rubber on dry glass

**Figure 2:**
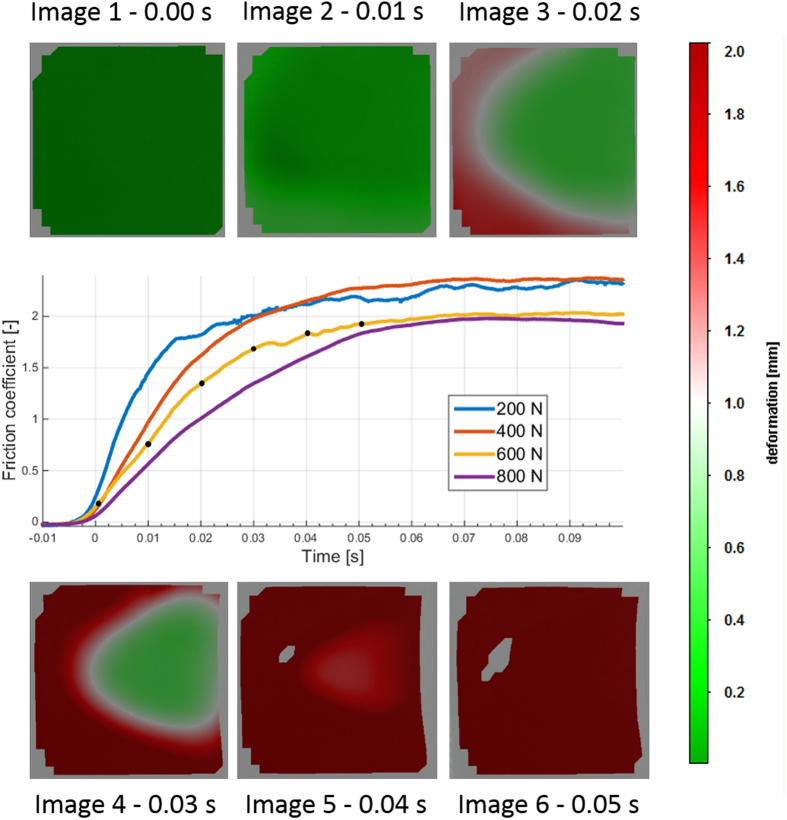
Detachment process of rubber on dry glass. The rubber block is moving to the right. The line figure shows four different load cases, and the images show the load case of 600 N (black dots indicate image snapshot). An actual high-speed photograph is at the background of the image and overlaid by a colour map to indicate local movement. A video is provided in the supplementary material.

Figure 2 shows how a static rubber block is detached to commence sliding. Force curves are given for different loads, and an image sequence is shown for a 600 N load (0.166 N/mm^2^). The detachment is clearly initiated from the rear corners of the contact area (image 3, 0.02 s) and continued by a detachment from the sides and rear (image 4, 0.03 s). The last area to stick to the glass is the middle area of the rubber after the detachment of the leading edge (images 4–5, 0.03–0.05 s). Complete detachment is observed after 0.05 s, where the force curve shows convergence into steady sliding friction as well.

The beginning of the force curve indicates a smooth transition from the static situation to sliding without any identifiable peak value that might be considered μ_s_. In addition, the force ramp shows no precursors of sliding when sampled at 10 000 Hz. The friction coefficient is clearly smaller for the 600 N and 800 N loads, indicating the expected load dependency. All loads and repetitions showed similar detachment patterns. For one of the several test sessions, the sample was slightly aligned with the glass surface, which resulted in a detachment from the other rear corner. However, the final sticking area was still in the middle of the contact.

The complete detachment time clearly depends on the load; however, the exact instant of time for achieving complete global sliding is difficult to identify (from either the force data or the image data).

### Rubber on wet glass

**Figure 3:**
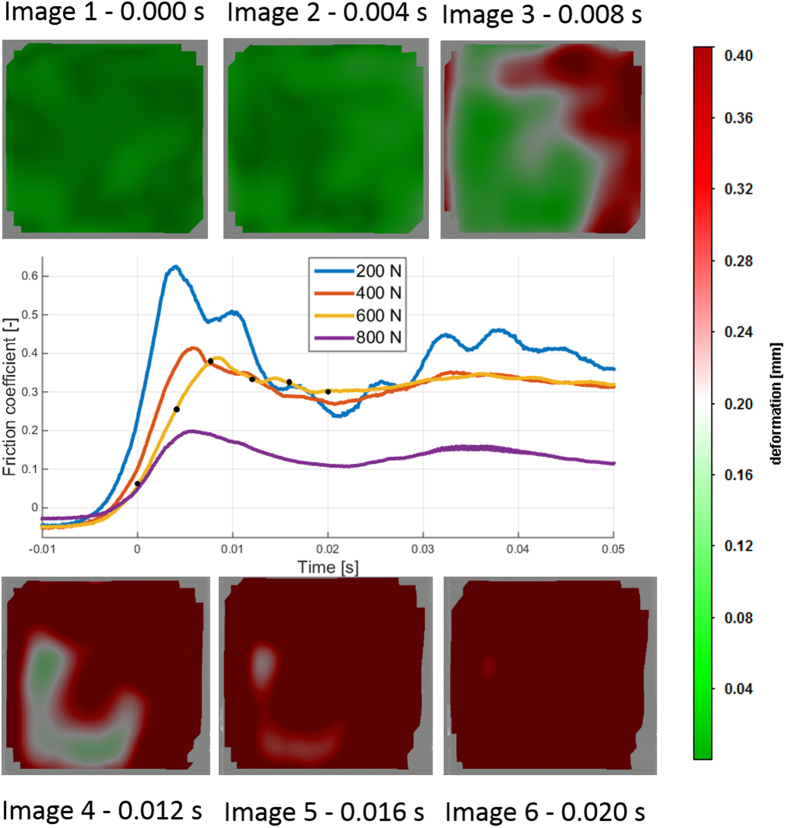
Detachment process of rubber on wet glass for four different load cases. Video is provided in the supplementary material.

Figure 3 shows the detachment propagation on wet glass (with similar test parameters as in the case of the dry glass). The DIC image sequence shows how the contact is very abruptly ripped to enable sliding within a few milliseconds. The complete contact area is already sliding at 0.016 s, whereas the same sample has not started clear detachment on dry glass (Fig. 2). The detachment pattern on wet glass is very chaotic, and even from the raw image data (not shown), identifying a detachment pattern similar to that observed in the case of the dry glass is not possible.

The force curve shows a clear peak value, which would traditionally be the μ_s_. The force ramp does not show clear precursors, but they are occasionally observed in the measurement setup and more often for the wet surface than for the dry. An interesting feature is observed when comparing images 1 and 2. Image 1 shows randomly distributed spots, which is noise due to the numerical analysis of the digital image detector data (see supplementary material video for noise properties). In the 4 ms image, where one cannot state that the contact at this length scale would slide, the image colour is more even and deterministic, indicating micro-slip and new settling of the sample under shear loading. Similar micro-movement is observed for the dry friction between images 1 and 2. This result indicates that the time sequence of images at one length scale is not sufficient to judge whether a contact is locally sliding or not, as sliding may already occur at some shorter length scale. Thus, the length scale and even multi-scale surface roughness is essential when defining local sliding motion. This shear-induced micro-movement might explain the velocity strengthening of the static friction^19^, which is an important but often overlooked phenomenon.

### Effect of dwell time

**Figure 4:**
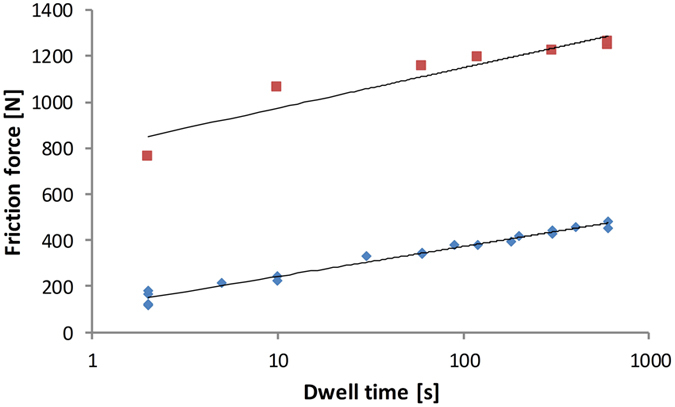
Influence of dwell time on static friction force on dry and wet surfaces.

The μ_s_ is known to be sensitive to the dwell time before the actual movement^20^,^21^,^22^,^23^,^24^, which is very likely from the increasing real contact area as a function of the contact time. The effect of the dwell time on the static friction was studied in the linear friction tester by loading the rubber sample against a dry or wet glass surface with a force of 400 N for different dwell times ranging from 2 to 600 s. Figure 4 confirms that the dwell time substantially influenced the static friction force on dry glass. A similar tendency is observed for the lubricated case (wet glass). In particular for the wet glass, the μ_s_ is almost three times higher for a 10 min dwell time than for the 2 s dwell time. The effect of the dwell time on the dry glass is of a similar force magnitude to that on the wet glass, although it clearly represents a smaller percentage (only a 50% increase from 2 s to 100 min, whereas on wet glass, the μ_s_ doubles). Interestingly, the rubber static friction depends strongly on the dwell time even on an ice surface^25^,^26^. Thus, the strong dwell time effect is very much due to the visco-elasticity of the rubber material^27^. The rubber is creeping into surface roughness, which consequently increases the real contact area when the dwell time increases^28^.

### FEM-simulation results

Finite element method (FEM) simulations were performed to study the initial shear force development due to vertical and shear loading. We did not intended to simulate the extremely complex sliding friction, but rather the phases prior to it. Unlike the models used in many other studies, the model used here describes the local friction law in a very simple way (Amontons’ law) but the bulk material properties are modelled by strain energy functions that are realistic for incompressible materials. This model provides a clear advantage over models consisting of masses and springs, where local contacts are described in a more complex but empirical way.

**Figure 5:**
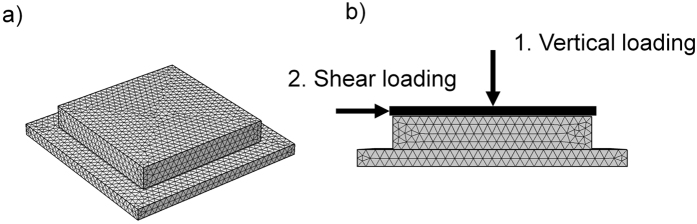
(**a**) The 3-dimensional FEM geometry with meshing; the upper block is the rubber sample and the lower block is a rigid and fixed surface. (**b**) The rubber sample (in the middle) is loaded vertically in steps by the prescribed displacement, and the shear loading is subsequently applied in steps. The displacements are acting on the top surface of the rubber sample.

A rubber sample (60 × 60 mm^2^) was pressed against a flat glass surface with a boundary condition similar to that used in the experiments (prescribed displacement of top surface). The vertical load was applied to the rubber sample in small steps to obtain a realistic initial shear stress development and distribution. After loading, a longitudinal movement was applied to the top of the sample, also stepwise. The loading conditions and meshing are shown in Fig. 5.

**Figure 6:**
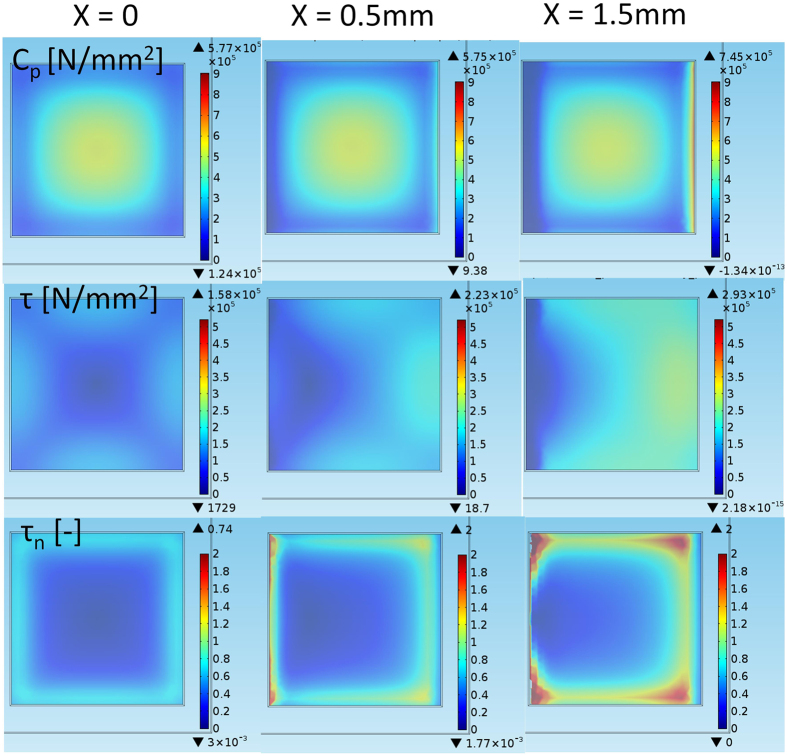
High-friction (μ = 2) detachment. The first, second, and third rows show the contact pressure C_p_ for the increasing longitudinal movement X, the shear stress τ, and the normalized shear stress 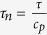, respectively.

Figure 6 shows the detachment pattern for the high-friction situation, e.g., a rubber-glass material pair, as in the experimental section of this paper. The figure shows the contact pressure C_p_, shear pressure τ and normalized shear pressure 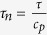 in the respective rows and the longitudinal movement steps in the columns. The contact pressure is concentrated in the middle part of the sample, and the shear stresses are concentrated near the sample edges. The shear stress is naturally zero in the middle of the sample because of symmetry. The contact pressure at the leading edge increases substantially, necessitating an increasing shear stress to originate detachment. By contrast, the contact pressure decreases substantially at the trailing edge of the sample, and, even if initial movement decreases, the shear stress at the trailing edge (when the direction of the initial shear stress is opposite that of the sliding, as illustrated in the discussion section) during the very first moments before global sliding, the detachment initiates from the rear corners of the sample, as observed in the normalized shear stress development. The local detachment is initiated when the normalized shear stress exceeds the local friction potential (μC_p_(x,y)). Poisson expansion due to vertical loading does not cause any local sliding, as *τ*_*N *_< *μ* (missing red colour tones (*τ*_*N*_) in case of x = 0).

**Figure 7:**
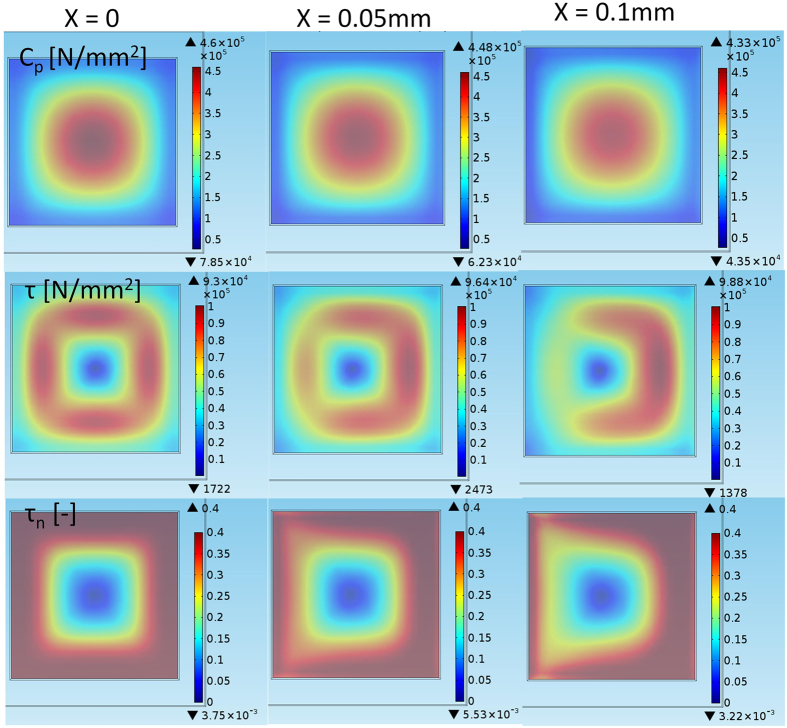
Low-friction (μ = 0.4) detachment. The first, second, and third rows show the contact pressure C_p_ for the increasing longitudinal movement X, the shear stress τ, and the normalized shear stress 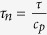, respectively.

A substantially different initial shear stress distribution is observed for a low-friction surface, as shown in Fig. 7. Additionally, the contact pressure is more evenly distributed than on dry glass. This result is explained by local sliding due to the loading of the sample: the restriction of a slippery surface to sliding is smaller than that on a high-friction surface. That is, the sample can deform to a greater extent and more freely against the low-friction surface, and the initial shear stress level is lower (than on a high-μ surface). A similar phenomenon is observed for rolling tyres, where the contact length is slightly longer on an icy surface, which can be used to identify road friction conditions^29^. When a sample begins to move, additional shear stresses are introduced; however, because the peak stresses are missing, the detachment to sliding is more abrupt and chaotic. Interestingly, because all contact regions can be utilized to prevent sliding, the static friction is higher than if a spotwise shear stress peak would trigger a detachment wave in the early phase of shear stress development. In this simulation scenario, the moment bending of the sample is smaller on the low-friction surface; thus, the contact pressure is not altered as much as in the case of the high-friction surface. This situation actually creates an opportunity to control the μ_s_/μ_d_ ratio by changing how the force acts on the sample^1^, and the static friction can be modified without changing the contacting materials.

**Figure 8:**
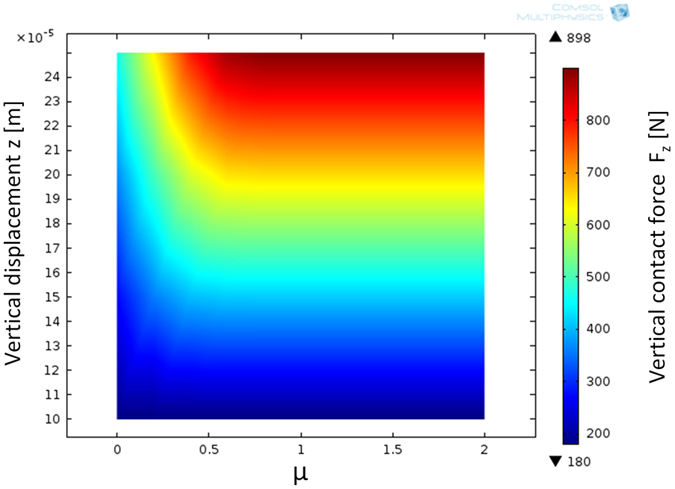
Vertical contact force F_z_ as a function of the friction coefficient and vertical displacement z under compression (from FEM). The figure indicates the strong interconnection of the sample stiffness (ΔFz/Δz) and μ on slippery surfaces (μ < 0.5).

The previous figures indicate that in addition to the shear pressure, the friction coefficient also affects the contact pressure distribution. Basically, on a slippery surface, the bulk rubber can expand laterally to a greater degree because the friction does not restrict this motion. For an incompressible material, the friction also has a strong influence on the vertical stiffness of the sample, i.e., the sample stiffness in compression is not purely a material property. This situation is illustrated in Fig. 8, where a rubber sample is pressed against the surface, with μ varying from 0 to 2. The colour indicates the vertical force acting on the sample. For μ greater than 0.5, the ΔFz/Δz ratio is constant because the sample is fully stuck to the surface, and an increase in μ does not actually change the contact conditions. However, for slippery μ values (below 0.5), a strong dependency of μ on the sample stiffness appears, e.g., the sample is softer on a low-μ surface. Thus, the material’s Poisson’s ratio affects the bulk material stiffness in terms of the friction as well. As a consequence, the tensile testing of materials tends to better reflect bulk material properties, whereas compressive testing is more dependent on the measurement setup. Naturally, in terms of the contact mechanics and friction, the material properties in compression are important.

## Discussion

**Figure 9:**
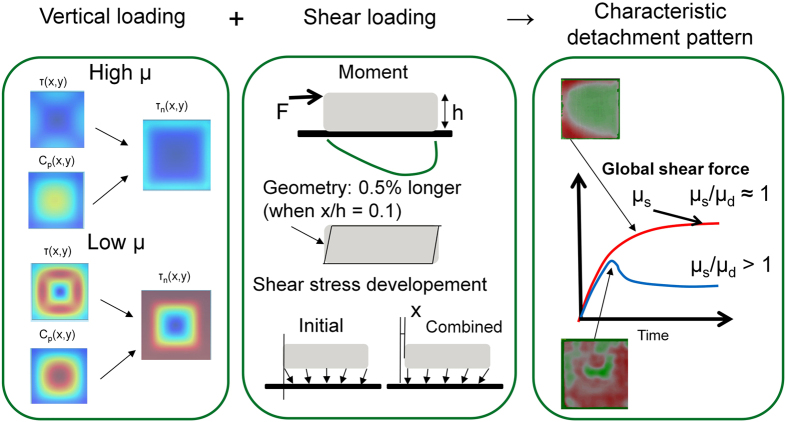
Key factors affecting the characteristic detachment pattern.

Figure 9 collects the experimental and simulation results of this work and combines them, showing the physical features that cause specific detachment patterns. On the left side of the figure, two finite element method (FEM) simulations of rubber settling on a perfectly smooth surface are shown. In the first scenario, the rubber-surface friction coefficient is 2, reflecting a dry surface μ_s_ for the rubber. Contact deformation results in a shear force distribution where the peaks (4) are located halfway between the corners at the edge of the sample. The contact pressure maximum is at the centre because of the incompressibility of rubber. The normalized shear force distribution shows peaks at the points where the local sliding is prone to initiate. The global frictional detachment naturally requires a tangential force to be applied to the sample; here, we assume that a force is acting on the top of the sample (if the force is applied near the contact, the behaviour would be different)^1^. The tangential loading introduces a moment that decreases the trailing edge contact pressure and finally triggers sliding friction near the trailing edge corners. This process would explain the experimental results observed in this work and gives a μ_s_ that is similar to μ_d_.

In the case of diminishing friction between the slider and surface (Fig. 9, Low μ), the initial tangential shear stresses cannot develop as in the previous case. Notably, no significant shear stress is observed at the edges because of the initial sliding. Additionally, the resulting contact pressure distribution is slightly different and the apparent contact area is larger because of the expansion of the bulk material. The missing shear stress peaks do not provide a natural singularity for slip propagation; thus, the onset of frictional sliding is chaotic and coincides everywhere in the contact. Furthermore, the moment arising from the lever arm of the applied force does not cause the trailing edge to initiate slip propagation. However, the overall break-away force is less than for the “dry” case; thus, the contribution of the moment is weaker. If the initial shear stresses due to loading could be smoothened, e.g., by structural design, the moment might become a dominant factor in slip initiation even for slippery surfaces, and μ_s_/μ_d_ would decrease.

In addition to the effect of the moment lever arm on detachment, some other mechanisms are also present because of shear loading. The middle image (shear loading) illustrates how the leading and trailing edges are actually lifting off because of the modified geometry of the elastic block. The magnitude is small compared to the sample size, but it substantially affects the local vertical force^30^. The friction and Poisson expansion together cause an initial shear stress field, as demonstrated earlier in this paper. However, when global shear loading is applied to the sample, this initial distribution is modified. Indeed, the shear stress at the leading edge is increased because the global shear loading is pointing in the same direction as the initial shear stress (lowest image in shear loading). An opposite effect exists for the trailing edge, and this contribution would suggest detachment from the leading edge (as for the rubber ice precursor)^25^. However, other factors normally dominate and lead to different detachment patterns.

Poisson expansion results in an initial shear stress field^31^,^32^, which appears to dominate the onset of the frictional sliding of rubber together with the loading conditions. The bulk properties of rubber cause sliding friction on rough surfaces^4^. On the basis of these observations, the bulk properties, especially the Poisson expansion, dominate the static friction coefficient or at least the μ_s_/μ_d_ ratio (when loading conditions are similar). This result may open new opportunities for industrial applications for rubber seals and automotive tyres: not only surface texturing and patterns have an effect, but the bulk properties and, e.g., chambers inside materials could also be used to modify μ_s_. The static friction can be modified by the timing of the micro-slip regions^33^ and could be used, e.g., to improve the grip of shoes^34^.

Precursors of slip are sometimes observed for wet glass–rubber contact and for soft rubber with a tire tread pattern on dry glass^35^. The precursors indicating that the μ_s_/μ_d_ relation is large (for a macroscopic obstacle) would be a convenient conclusion, but there is no generic relation. The dry surface friction shows no precursors because the initially uneven shear stress distribution provides clear nucleation points for sliding. The local sliding motion then propagates smoothly and in a deterministic manner. By contrast, the lubrication provided by the water between the rubber and glass smooths the initial shear stresses, resulting in no strong candidate locations for the nucleation of the sliding motion. Consequently, the detachment of the rubber to initiate sliding is very abrupt. Consequently, the static friction becomes very large because μ_s_ is fully spatially utilized before transferring to μ_d_ (high local μ before detachment).

As a curiosity, Schallamach waves^36^ are never observed in our linear friction tester. A hemispherical slider and very low sliding velocities are apparently needed to create a suitable condition for such buckling. Meanwhile, a normal stick slip is often observed, as reported in detail elsewhere^35^. However, stick-slip (and friction in general)^1^ is very much a tribo-system-specific property, and contacting materials are just one aspect of it.

Many authors^2^,^20^,^23^,^37^,^38^ have reported that that the static friction coefficient of rubber depends on the initial dwell time and the rate of starting, which is definitively consistent with our results. Here, this effect is even stronger under lubricated conditions. The effect of the dwell time in the lubricated condition is critical in, e.g., hydraulic cylinders, where long standstill times and large differences in potential break-away forces make the control system design challenging. The dwell time has at least the following effects on contact: 1. Rubber creep increases the real contact area (increase in μ_s_) 2. Creep results in smoothening of the initial shear stress^12^ (increase in μ_s_) 3. The trapped air/lubricant slowly bleeds away from the contact (increase in μ_s_) 4. Formed capillary bridges pull surfaces together. All these contributions increase the static friction force. As a practical case, the strong effect of the dwell time on the break-away force makes, e.g., accurate control of a hydraulic cylinder more difficult; however, the problem could be solved by the advanced engineering of seals. The ideal design of a ring-type rubber seal could have a pattern that without causing any leakage, would allow a natural point for a frictional shear crack to develop and let it propagate deterministically through the contact area. The strong load dependency of the friction of rubber and some other materials might be linked to Poisson expansion because strong local stresses tend to decrease (static) friction.

On the basis of both the experiments and simulations presented in this paper, we conclude that detachment propagates in 2D and that consequently, 1D measurements cannot reveal the underlying phenomena (as reported elsewhere)^10^. To fully understand the onset of frictional sliding in three dimensions, the micro-slip and roughness at several length scales must be considered. Furthermore, a high-fidelity bulk material simulation model is equally or even more important than an interface friction model to predict and understand μ_s_. The local friction might even be modelled by Amontons’ law, whereas the bulk material and loading properties transfer these local friction forces into non-Amontons’ behaviour of a macroscopic slider.

## Methods

### Experiments

A linear friction tester at Aalto University was used to study the rubber detachment process in this work^39^,^40^. The rubber sample was glued into an alloy sample holder that was mechanically fixed to the piezoelectric force sensors. The force data were acquired at 10 000 Hz. The force sensors were connected to a pneumatic cylinder with a digital control valve, which applied vertical force to the sample. The longitudinal movement was constrained by a control arm, which allowed the vertical movement of the force sensor and sample. These components were in a carrier that was fixed to the linear guide, allowing longitudinal motion for friction studies. The longitudinal motion was controlled with a servo motor.

The sliding surface was a glass plate that could be moved laterally. Thus, the glass surface position for the sample could be changed without moving the high-speed camera. The high-speed camera (Photron SA-3) was equipped with a 50 mm focal-length objective. The spatial resolution of the optical setup was approximately 0.08 mm, and the frame rate was 5000 Hz.

**Figure 10:**
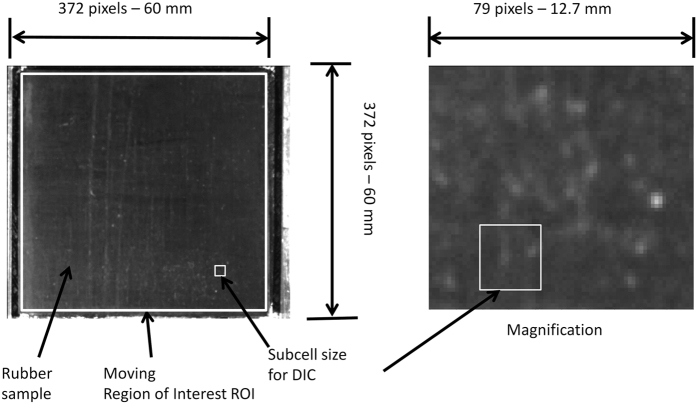
A rubber sample viewed through the glass plate with a high-speed camera. The DIC parameters are shown.

The camera was calibrated to the metric scale by capturing an image in which a ruler was laying on the glass. The image sequence was analysed with digital image correlation (DIC, Fig. 10), where two consecutive images were compared to identify the deformation field of the image. In particular, the images were divided into smaller sub cells, and the displacement (x and y) giving the maximum cross correlation was determined to be the deformation. Typically, the sub-cell size in these analyses was 79 × 79 pixels. The region of interest where the deformations were calculated was able to move on the basis of the deformation history. The grey-scale resolution was 12-bit.

The local velocities were calculated from the displacement field and were illustrated in this paper by overlaying a colour indicating the displacement over the actual image.

The target sliding speed of the sample was 0.2 m/s, and a servo motor applying longitudinal motion provided 8 m/s^2^ target acceleration. The force sensors also measured the inertial forces (of magnitude of a few newtons) in addition to the friction forces when the rubber sample was accelerating. The sample weight was removed from the force signal. The rubber sample was a 60 mm × 60 mm × 10 mm solid rubber block without any tread pattern or texture. No additional markers were required for the DIC analysis.

### Simulation model

The finite element method model was implemented in Comsol Multiphysics 5.0 using the solid mechanics interface and a stationary study. The model was run by forcing the top surface to move as a function of the vertical displacement parameter; after loading, the top surface was similarly moved longitudinally. The local friction force followed Amontons’ law. The model consisted of 39 720 tetrahedral elements, with an average element quality of 0.7646 and a minimum element quality of 0.2445. The strain energy function of the Yeoh incompressible material model is given by


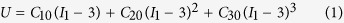


and the model parameters are *C*_10_ = 0.906261, *C*_20_ = −0.304 126, and *C*_30_ = 0.082763 for the tire-tread rubber compound described by Cai *et al*.^41^. The counter surface was rigid and smooth.

## Additional Information

**How to cite this article**: Tuononen, A. J. Onset of frictional sliding of rubber-glass contact under dry and lubricated conditions. *Sci. Rep.* **6**, 27951; doi: 10.1038/srep27951 (2016).

## Supplementary Material

Supplementary Information

Supplementary Video for Figure 2

Supplementary Video for Figure 3

## References

[b1] Ben-David O. & Fineberg J. Static Friction Coefficient Is Not a Material Constant. Phys. Rev. Lett. 106, 1–4 (2011). 10.1103/PhysRevLett.106.254301 21770644

[b2] Persson B. N. J. . On the nature of the static friction, kinetic friction and creep. Wear 254, 835–851 (2003).

[b3] Capozza R. & Urbakh M. Static friction and the dynamics of interfacial rupture. Phys. Rev. B 86, 085430 (2012).

[b4] Grosch K. The relation between the friction and visco-elastic propeties of rubber. Proc. Roy. Soc. A 274, 21–39 (1963).

[b5] Schallamach A. A theory of dynamic rubber friction. Wear 6, 375–382 (1963).

[b6] Klüppel M. & Heinrich G. Rubber Friction on Self-Affine Road Tracks. Rubber Chem. Technol. 73, 578–606 (2000).

[b7] Persson B. N. J. Theory of rubber friction and contact mechanics. J. Chem. Phys. 115, 3840 (2001). 10.1063/1.4936558 26671362

[b8] Mahboob Kanafi M., Kuosmanen A., Pellinen T. K. & Tuononen A. J. Macro- and micro-texture evolution of road pavements and correlation with friction. Int. J. Pavement Eng. 16, 168–179 (2015).

[b9] Lorenz B. . Static or breakloose friction for lubricated contacts: the role of surface roughness and dewetting. J. Phys. Condens. Matter 25, 445013 (2013). 24131947 10.1088/0953-8984/25/44/445013

[b10] Romero V., Wandersman E., Debregeas G. & Prevost A. Probing locally the onset of slippage at a model multicontact interface. Phys. Rev. Lett. 112, 1–5 (2014). 10.1103/PhysRevLett.112.094301 24655257

[b11] Rubinstein S. M., Cohen G. & Fineberg J. Detachment fronts and the onset of dynamic friction. Nature 430, 1005–1009 (2004). 15329715 10.1038/nature02830

[b12] Rubinstein S. M., Cohen G. & Fineberg J. Visualizing stick–slip: experimental observations of processes governing the nucleation of frictional sliding. J. Phys. D. Appl. Phys. 42, 214016 (2009).

[b13] Maegawa S., Suzuki A. & Nakano K. Precursors of Global Slip in a Longitudinal Line Contact Under Non-Uniform Normal Loading. Tribol. Lett. 38, 313–323 (2010).

[b14] Otsuki M. & Matsukawa H. Systematic breakdown of Amontons’ law of friction for an elastic object locally obeying Amontons’ law. Sci. Rep. 3, 1586 (2013). 23545778 10.1038/srep01586 PMC3613807

[b15] Trømborg J. K. . Slow slip and the transition from fast to slow fronts in the rupture of frictional interfaces. PNAS 111, 8764–8769 (2014). 24889640 10.1073/pnas.1321752111 PMC4066514

[b16] Nguyen T. . Surface pressure and shear stress fields within a frictional contact on rubber. J. Adhes. 87, 235–250 (2011).

[b17] Audry M. C., Fretigny C., Chateauminois A., Teissere J. & Barthel E. Slip dynamics at a patterned rubber/glass interface during stick-slip motions. Eur. Phys. J. E. Soft Matter 35 (2012). 10.1140/epje/i2012-12083-0 22972225

[b18] Prevost A., Scheibert J. & Debrégeas G. Probing the micromechanics of a multi-contact interface at the onset of frictional sliding. Eur. Phys. J. E, Soft matter 36 (2013). 10.1140/epje/i2013-13017-0 23456433

[b19] Bar-Sinai Y., Spatschek R., Brener E. A. & Bouchbinder E. Velocity-strengthening friction significantly affects interfacial dynamics, strength and dissipation. Sci. Rep. 5, (2015). 10.1038/srep07841 PMC4297976 25598161

[b20] Roberts A. D. & Othman A. B. Rubber adhesion and the dwell time effect. Wear 42, 119–133 (1977).

[b21] Roberts A. D. Rubber Adhesion Variation with Dwell Time: Influence of Polymer Type, Substrate and Environment. Adhesion 14, 51–70 (1990).

[b22] Kasem H. & Etsion I. Experimental study of the effect of dwell time and normal load on static friction in creeping elastic–plastic polymer spherical contact. Wear 309, 139–145 (2014).

[b23] Malamut S., Kligerman Y. & Etsion I. The Effect of Dwell Time on the Static Friction in Creeping Elastic–Plastic Polymer Spherical Contact. Tribol. Lett. 35, 159–170 (2009).

[b24] Brockley C. A. & Davis H. R. The Time-Dependence of Static Friction. J. Lubr. Technol. 90, 35–41 (1968).

[b25] Isomaa J.-M., Tuononen A. J. & Bossuyt S. Onset of frictional sliding in rubber–ice contact. Cold Reg. Sci. Technol. 115, 1–8 (2015).

[b26] Schulson E. M. & Fortt A. L. Static strengthening of frictional surfaces of ice. Acta Mater. 61, 1616–1623 (2013).

[b27] Persson B. N. J., Albohr O., Creton C. & Peveri V. Contact area between a viscoelastic solid and a hard, randomly rough, substrate. J. Chem. Phys. 120, 8779–8793 (2004). 15267810 10.1063/1.1697376

[b28] Rubinstein S. M., Cohen G. & Fineberg J. Contact area measurements reveal loading-history dependence of static friction. Phys. Rev. Lett. 96, 1–4 (2006). 10.1103/PhysRevLett.96.256103 16907326

[b29] Tuononen A. J. Tire-Road contact information for Advanced driver Assistance systems: Optical tyre sensor approach. In Tire Technology International Annual Review 1–26 (UKIP Media & Events Ltd, 2009).

[b30] K. H., J. E. & H. A. M. A Thermo-Mechanical Formulation Describing the Frictional Behavior of Rubber. Proc. Appl. Math. Mech 2, 238–239 (2003).

[b31] Brörmann K., Barel I., Urbakh M. & Bennewitz R. Friction on a Microstructured Elastomer Surface. Tribol. Lett. 50, 3–15 (2013).

[b32] Ozaki S., Inanobe C. & Nakano K. Finite Element Analysis of Precursors to Macroscopic Stick–Slip Motion in Elastic Materials: Analysis of Friction Test as a Boundary Value Problem. Tribol. Lett. 55, 151–163 (2014).

[b33] Lorenz B. & Persson B. N. J. On the origin of why static or breakloose friction is larger than kinetic friction, and how to reduce it: the role of aging, elasticity and sequential interfacial slip. J. Phys. Condens. Matter 24, 225008 (2012). 22580928 10.1088/0953-8984/24/22/225008

[b34] Moriyasu K., Nishiwaki T., Yamaguchi T. & Hokkirigawa K. Experimental Analysis of the Distribution of Traction Coefficient in the Shoe-Ground Contact Area during Running. Tribol. Online 7, 267–273 (2012).

[b35] Tuononen A. J. Digital image correlation to analyse stick-slip behaviour of tyre tread block. Tribol. Int. 69, 70–76 (2014).

[b36] Schallamach A. How does rubber slide. Wear 17, 301–312 (1971).

[b37] Kendall K. Rolling friction and adhesion between smooth solids. Wear 33, 351–358 (1975).

[b38] Tuononen A. J. Static friction coefficient of rubber on dry and wet glass : Influence of dwell time. in Wear of Materials (Elsevier, 2013).

[b39] Rantonen M., Tuononen A. J. & Sainio P. Measuring stud and rubber friction on ice under laboratory conditions. Int. J. Veh. Syst. Model. Test. 7, 194–207 (2012).

[b40] Fülöp T. & Tuononen A. J. Evolution of ice surface under a sliding rubber block. Wear 307, 52–59 (2013).

[b41] Cai Y., Zang M., Chen Y. & Liu W. Experiments and finite element simulations of a tyre blow-out process. Proc. Inst. Mech. Eng. Part D J. Automob. Eng. 228, 1116–1124 (2014).

