# Supplementary material for: Onset of frictional sliding of rubber–glass contact under dry and lubricated conditions
Source: Sci Rep. 2016 Jun 13;6:27951. doi: 10.1038/srep27951 (PMC4904280; doi:10.1038/srep27951)
Supplement: Supplementary Information [file srep27951-s1.pdf]

Supplementary file information:

Onset of frictional sliding of rubber–glass contact under dry and lubricated conditions

Ari J. Tuononen

Aalto University

Department of Mechanical Engineering

PO Box 14300

FI-00076 AALTO

FINLAND

Tel. +358 50 5604702

ari.tuononen@aalto.fi

Supplementary file information:

Video 600N_Dry_01.avi:

Video for Figure 2: Detachment process of rubber on dry glass. Vertical load 600N.

Video 600N_Wet_01.avi:

Video for Figure 3: Detachment process of rubber on wet glass. Vertical load 600N.
